# Incremental validity of acceptance over coping in predicting adjustment to endometriosis

**DOI:** 10.3389/fpain.2022.928985

**Published:** 2022-07-15

**Authors:** Olivia Bernini, Giovanni Tumminaro, Lisa Compare, Cristina Belviso, Valentina Conforti, Carmen Berrocal Montiel

**Affiliations:** ^1^University Counseling Services, University of Pisa, Pisa, Italy; ^2^Department of Surgical, Medical and Molecular Pathology and Critical Care, University of Pisa, Pisa, Italy; ^3^Associazione Progetto Endometriosi, Parma, Italy

**Keywords:** psychological acceptance, coping, chronic pain, endometriosis, psychological distress

## Abstract

Psychological acceptance has emerged as an important construct to explain low psychological distress in different clinical samples. However, the incremental validity of psychological acceptance to explain adjustment to medical conditions over other related and well-established constructs, such as coping, is relatively unclear. This study explored whether psychological acceptance significantly contributes to explain adjustment above and beyond coping in females with endometriosis. A total of 169 females (*M*_*age*_ = 34.95 years; *SD*_*age*_ = 6.07 years) with endometriosis and pain symptoms completed the Acceptance and Action Questionnaire-II, the Brief-COPE, the Hospital Anxiety and Depression Scale, the Psychological Wellbeing Scale, and the Endometriosis Health Profile-5. We conducted Hierarchical Regression Analyses to determine the contribution of psychological acceptance to explaining adjustment. The results showed that the contribution of psychological acceptance ranged from 11 to 20% when controlling for coping, while coping explained from 1 to 8% when the model was reversed. The findings suggest that psychological acceptance is a more useful construct than coping for predicting PD and other psychological outcomes in females with endometriosis.

## Introduction

Endometriosis is a gynecological condition characterized by the presence of ectopic endometrial tissue outside the uterus (1). This disease affects up to 10% of women in the general population (2, 3), and occurs in about 40%−60% of women with pelvic pain (2). Endometriosis is mainly associated with infertility and with a range of pain symptoms, including dysmenorrhea, dyspareunia, dyschezia, lower back pain, and chronic pelvic pain (2, 4, 5).

Previous findings showed that pain is one of the main symptoms contributing to impaired health-related quality of life (HRQoL) in women with endometriosis [e.g., (6–10)]. Furthermore, previous research found that psychological distress was higher in women with endometriosis and illness-related pain (i.e., endometriosis-related pain) when compared to healthy women (11), women with benign gynecological symptoms (12), and women with asymptomatic endometriosis (11–13).

Pain acceptance has emerged as an important process to explain interindividual differences in adjustment to chronic pain (14–16). Pain acceptance consists of two related processes: willingness to experience pain, and engaging in life activities regardless of pain (16). Research showed that pain acceptance prospectively predicts emotional, physical and social functioning, and less psychological distress in patients with chronic pain [e.g., (17–20)]. Moreover, pain acceptance proved to have incremental validity over and above pre-existing predictors, such as coping strategies (21, 22), to explain adjustment to pain (23–25). Pain acceptance-based interventions (i.e., Acceptance and Commitment Therapy, ACT) (26, 27) demonstrated to significantly improve patients' functioning and to reduce the utilization of health-care services [e.g., (28–32)].

While there is substantial evidence indicating pain acceptance as a protective factor for a positive adjustment to chronic pain, few studies have investigated the contribution of general psychological acceptance to pain-related medical conditions. General psychological acceptance refers to the willingness of people to experience difficult inner events (e.g., negative feelings, memories, thoughts), and to engage in valued actions even in the presence of such internal experiences (33, 34). Hence, compared to pain acceptance, general psychological acceptance may allow patients to accept not only pain but also other difficult internal events such as unwanted thoughts and feelings (35). Psychological acceptance showed significant associations with a wide range of outcomes, including higher quality of life and life satisfaction, lower depression, anxiety and other mental-health criteria across different clinical and nonclinical populations (34, 36, 37).

In the context of chronic pain, McCracken & Zhao O'Brien (35) showed that higher psychological acceptance had a significant and unique contribution in explaining better functioning over background variables, pain, pain acceptance and mindfulness in a sample of adult patients, with a 2%−9% increase of explained variance across various outcomes. Moreover, McCracken & Velleman (38) found that psychological acceptance accounted for an average of 24% of variance in predicting health status and general practitioner visits related to pain, independently of background variables, pain acceptance and mindfulness, in a sample of patients attending primary care settings.

Whether the above findings generalize to nonclinical samples of people suffering from pain is still largely unexplored. Moreover, the incremental validity of psychological acceptance over well-established predictors of adjustment to illness, such as coping, has not been proved yet. Overall, research found that problem-focused and detached coping strategies relate to positive outcomes (9, 39, 40), whereas coping strategies mainly based on avoidance and suppression of emotions are associated with increased pain and psychological distress (9, 39, 41–43). Hence, while coping seems to be an important predictor of different outcomes in endometriosis, whether psychological acceptance improves the prediction of adjustment, over and above coping, is still unknown.

The present study was aimed at analyzing the criterion validity of psychological acceptance to explain adjustment in a sample of females with endometriosis recruited in a non-clinical setting. In line with previous acceptance-related work, we hypothesized the contribution of psychological acceptance to explain adjustment criteria to be significant, with higher psychological acceptance predicting lower depression, anxiety, and disability, and higher psychological wellbeing. This study also extends previous related work by evaluating the incremental validity of psychological acceptance in explaining adjustment to endometriosis over and beyond coping. We hypothesized that psychological acceptance can improve the prediction of depression, anxiety, disability, and psychological wellbeing beyond measures of coping strategies.

## Materials and methods

### Participants

Participants were recruited through the Italian Association for the Endometriosis Project (AEP). The AEP is a social, no-profit organization whose members are females with endometriosis. A total of 169 females with endometriosis and related-illness pain participated in the study. The sample consisted of ~17% of the AEP members.

The mean age of the sample was 34.95 years (SD = 6.07, range = 18–56). Around 67.5% of the sample was married or in a stable relationship and the remaining 32.5% was single, divorced or widow. Half of the sample (50%) had completed primary or secondary schools, and the remaining half had a University degree. Most participants (80.5%) were full- or part-time employed. The average duration of pain (from the diagnosis of endometriosis) was 73.11 months (*SD* = 64.80).

### Procedure

The present study was conducted in accordance with the Declaration of Helsinki, and approved by the AEP Board. AEP members were approached *via* e-mail through AEP Scientific Board. Females with endometriosis, illness-related pain, and aged 18 years or older were invited to participate in the study. Participants did not receive any remuneration for participating in the study. Females who provided informed consent to participate were asked to complete online the assessment described below. Participants received an anonymous code to access the questionnaires. The link to the questionnaire package was available on the AEP website for a month. Online questionnaires were set up to force respondents to answer all questions.

### Measures

The main variables in the study were measured through self-report questionnaires.

The 7-item version of the *Acceptance and Action Questionnaire-II* (AAQ-II) (44, 45) was used to measure psychological acceptance. Items are rated on a 7-point Likert scale, ranging from 1 to 7. Higher scores indicate lower levels of acceptance. However, for the purpose of this study, the scoring of the AAQ-II was reversed, so that higher scores reflected greater acceptance. Previous findings supported a unidimensional structure for the AAQ-II items [e.g., (44, 45)]. The Italian version of the scale demonstrated good internal consistency, and adequate criterion and convergent validity in a general population sample (45). Cronbach's alpha in this sample was 0.88.

The *Brief COPE* (46) - Italian version by Conti (47) - was used to measure coping strategies. It was designed to measure three ways to cope with stressful events: problem-focused coping (i.e., strategies aimed at solving or actively dealing with stressful events, such as planning or using instrumental support), emotion-focused coping (i.e., strategies aimed at managing emotions associated with stressful events, such as using humor or emotional support), and dysfunctional coping (i.e., dysfunctional strategies, such as substance abuse, or strategies aimed at avoiding emotional distress and stressful events such as denial, behavioral disengagement or self-distraction). The questionnaire consists of 28 Likert items ranging from 1 to 4, with higher scores indicating greater usage of the corresponding coping strategies. Previous research supported the factorial structure and internal consistency of the Brief COPE (46, 48). Cronbach's alpha coefficients in this sample were 0.68 for the problem-focused dimension, 0.69 for the emotion-focused coping, and 0.58 for the dysfunctional coping.

The *Hospital Anxiety and Depression Scale* (HADS) (49, 50) was used to assess anxiety and depression. It consists of two subscales (with seven items each) that measure anxiety and depression, separately, in the last week. Items are rated on a 4-point Likert scale ranging from 0 to 3. Higher scores indicate higher depression and anxiety. The HADS is a reliable and valid tool for assessing depression and anxiety in clinical and nonclinical populations (49–51). In our sample, Cronbach's alpha coefficients for the anxiety and depression subscales were 0.84 and 0.73, respectively.

The 18-item version of the *Psychological Wellbeing Scale* (PWB) (52, 53) was used to assess eudaimonic wellbeing, which focuses on how well people perceive different aspects of their functioning, including self-acceptance, autonomy, purpose in life, environmental mastery, positive relationships, and personal growth (54, 55). Items are rated on a 6-point Likert-type scale ranging from 1 to 6, with higher scores indicating higher levels of PWB. The Italian version of the questionnaire demonstrated good test-retest reliability, and significant positive correlation coefficients among the subscales (53). Cronbach's alpha coefficient was 0.85 in our sample.

The *Endometriosis Health Profile-5* (EHP-5) (56) was used to measure the health-related quality of life in women with endometriosis. It consists of five items rated on a 5-point Likert scale ranging from 0 to 4 according to how much symptoms of endometriosis interfered with work, daily activities, mood and feelings during the last month. Higher scores on the EHP-5 indicate higher interference, and hence higher functional and psychosocial disability. The EHP-5 demonstrated good internal consistency, and adequate criterion validity in a sample of females with endometriosis aged between 17 and 58 years (56). Cronbach's alpha coefficient was 0.79 in this sample.

Together with the above questionnaires, participants also completed a further brief questionnaire providing background information, including socio-demographic variables (age, educational level, employment, and marital status), duration of pain (months from endometriosis diagnosis) and pain intensity in the last week. Pain intensity was assessed using a scale ranging from 1 (“no pain”) to 10 (“worst possible pain”).

### Statistical analyses

The statistical analyses were performed using the Statistical Package for Social Sciences (SPSS) version 20.0. Two sets of Hierarchical Multiple Regression (HMR) models were tested to explore the unique and combined contribution of psychological acceptance and coping strategies to predict each measure of adjustment. In the first set of HMR analyses, the three coping dimensions were entered first as a block and AAQ-II scores were entered subsequently. In the second set of HMR analyses, the order for entry was reversed (AAQ-II scores were entered first and then coping strategies were entered as a block). Together the two sets of HMR allowed us to explore which variable (acceptance or coping) accounts for the largest increment of unique variance in the outcome measures. Background variables significantly associated with outcome measures were entered in the first step of each equation.

## Results

### Preliminary analyses

First, the data set was analyzed to detect possible violations of the HMR assumptions. The percentages of cases with absolute standardized residual values greater than 2 ranged from 3.5% (*n* = 6 cases for anxiety) to 5.3% (*n* = 9 cases for EHP-5 and depression). The percentage of cases showing absolute values >2.5 ranged from 0% (for disability) to 1.1% (*n* = 2 for anxiety). Additionally, no case showed Cook's values >1, suggesting that no case in this sample had an unusual influence on the regression models. Furthermore, the normal plots of the regression standardized residuals showed that data for each outcome were normally distributed, and the plots of the standardized residuals against the standardized predicted values showed that the assumptions of normality, homoscedasticity and linearity were not violated (Supplementary Figures 1–12).

Table 1 shows Pearson's correlation coefficients and descriptive statistics for the main variables in the study. Pearson's coefficients for the relations between background (socio-demographic/clinical) and outcome variables ranged from 0.01 to 0.55, and most of them were weak (*r* ≤ 0.15) and did not reach statistical significance. Only work status and educational level did significantly correlate with measures of adjustment. Unemployment was associated with lower PWB (*r* = −0.20, *p* ≤ 0.5), and higher education levels significantly correlated with lower depression (*r* = −0.18, *p* ≤ 0.05) and disability (*r* = −0.16; *p* ≤ 0.05). Pain duration did not correlate significantly with dependent variables, whereas pain intensity showed weak to moderate correlations with anxiety, depression, and disability. Higher pain intensity significantly correlated with higher depression (*r* = 0.27, *p* ≤ 0.001), anxiety (*r* = 0.30, *p* ≤ 0.001) and disability (*r* = 0.55, *p* ≤ 0.001).

**Table 1:** Pearson's correlation coefficients and descriptive statistics for the main variables in the study (*N* = 169)^+^.

**Variables**	**1**	**2**	**3**	**4**	**5**	**6**	**7**	**8**	**9**	**10**	**11**	**12**	**13**	**14**
1. Age	–													
2. Employment^#^	−0.14	–												
3. Educational level	−0.12	−0.08	–											
4. Marital status^∧^	−0.22^**^	−0.02	0.03	–										
5. Pain duration	0.53^***^	0.16^*^	−0.15^*^	−0.14	–									
6. Pain intensity	0.04	0.15^*^	−0.04	−0.08	0.15	–								
7. Acceptance	0.08	−0.04	0.10	−0.06	−0.02	−0.07	–							
8. Problem-focused coping	0.15^*^	−0.10	0.08	0.07	0.08	0.01	0.16^*^	–						
9. Emotion-focused coping	0.22^**^	−0.08	−0.11	−0.03	0.09	−0.14	0.22^**^	0.52^***^	–					
10. Dysfunctional coping	−0.07	0.08	0.02	0.11	−0.03	0.06	−0.53^***^	−0.05	−0.03	–				
11. Anxiety	−0.11	0.04	−0.12	−0.06	0.01	0.30^***^	−0.64^***^	−0.21^**^	−0.31^***^	0.39^***^	–			
12. Depression	−0.04	0.11	−0.18^*^	−0.05	−0.03	0.27^***^	−0.57^***^	−0.27^***^	−0.32^***^	0.28^***^	0.63^***^	–		
13. Disability	−0.11	0.15	−0.16^*^	−0.06	0.01	0.55^***^	−0.51^***^	−0.15	−0.18^*^	0.34^***^	0.58^***^	0.54^***^	–	
14. Psychological Wellbeing	0.14	−0.20^*^	0.09	−0.01	0.05	−0.05	0.62^***^	0.37^***^	0.36^***^	−0.36^***^	−0.56^***^	−0.65^***^	−0.44^***^	–
**Mean [Median]**	34.9	0.20	4.4	0.33	73.1	[4.0]	23.6	19.1	26.7	25.9	8.5	5.2	12.8	76.8
**SD**	6.1	0.40	1.6	0.57	64.8	2.6	10.4	3.2	5.1	4.5	4.2	3.3	3.6	14.2
**Range**	18–56	0–1	2–7	0–1	1–336	1–10	7–49	10–24	14–39	14–39	0–18	0–14	5–20	40–108

+ *N = 167 for duration of pain*.

# *Dummy variable: 0 = employed; 1 = unemployed*.

∧ *Dummy variable: 0: spoused/stable relationship, 1 = single*.

* *p ≤ 0.05*.

** *p ≤ 0.01*.

*** *p ≤ 0.001*.

Pearson's correlation coefficients for the relations between acceptance and outcome measures were moderate (ranging from 0.51 to 0.64) and statistically significant (*p* ≤ 0.001 for all coefficients). Higher acceptance significantly correlated with higher PWB and lower anxiety, depression and disability. All coefficients for the relations between coping and adjustment measures were weak (ranging from −0.15 to 0.39) and statistically significant. The only exception was the correlation between problem-focused coping and disability, which did not reach statistical significance. Both emotion-focused and problem-focused coping strategies correlated negatively with anxiety, depression and disability, and positively with PWB. Dysfunctional coping strategies positively correlated with anxiety, depression and disability, and negatively with PWB.

Correlations between each possible pair of predictors (acceptance, problem-focused coping, emotion-focused coping, dysfunctional coping) to be included in each HMR model were weak (ranging from −0.03 to 0.22). The only exceptions were the moderate correlations between acceptance scores and dysfunctional coping (*r* = −0.53, *p* ≤ 0.001), and between problem- and emotion-focused coping (*r* = 0.52, *p* ≤ 0.001).

### HMR models: Contribution of acceptance to explain adjustment (controlling for coping scores)

Table 2 shows the results from the first set of HRM analyses in which coping strategies were entered as a block before acceptance. Background variables accounted significantly with a 4% (for PWB) to 33% (for disability) of the variance in the outcome measures. In particular, pain intensity explained 7, 9, and 30% of variance in depression, anxiety, and disability, respectively. In the last step of each model, pain intensity proved to be a significant predictor for these outcomes. Higher pain intensity predicted higher anxiety (*t* = 4.16, *p* ≤ 0.001), depression (*t* = 3.45, *p* ≤ 0.001) and disability (*t* = 9.57, *p* ≤ 0.001) in the last step of each model.

**Table 2:** HMR models to predict adjustment (when coping is entered before acceptance).

**Criteria**	**Step**		***B***	**β**	***t***	***F***	**Δ*R*^2^**	**F *change***
**Anxiety**	**1**	Pain intensity	0.47	0.30	3.99^***^	15.90^***^	0.09	
	**2**	Pain intensity	0.39	0.24	3.65^***^	17.48^***^	0.21	16.53^***^
		Problem-focused coping	−0.11	−0.08	−1.05			
		Emotion-focused coping	−0.19	−0.23	−2.94^**^			
		Dysfunctional coping	0.34	0.36	5.49^***^			
	**3**	Pain intensity	0.38	0.23	4.16^***^	32.70^***^	0.20	65.91^***^
		Problem-focused coping	−0.08	−0.06	−0.90			
		Emotion-focused coping	−0.11	−0.13	−1.94			
		Dysfunctional coping	0.07	0.08	1.16			
		Acceptance	−0.22	−0.55	−8.12^***^			
**Depression**	**1**	Educational level	−0.37	−0.18	−2.35^*^	5.51^*^	0.03	
	**2**	Educational level	−0.35	−0.17	−2.27^*^	9.12^***^	0.07	12.36^***^
		Pain intensity	0.33	0.26	3.52^***^			
	**3**	Educational level	−0.39	−0.19	−2.79^**^	12.34^***^	0.18	13.15^***^
		Pain intensity	0.27	0.21	3.09^**^			
		Problem-focused coping	−0.12	−0.12	−1.51			
		Emotion-focused coping	−0.16	−0.25	−3.05^**^			
		Dysfunctional coping	0.19	0.26	3.86^***^			
	**4**	Educational level	−0.26	−0.13	−2.07^*^	20.67^***^	0.16	45.48^***^
		Pain intensity	0.27	0.21	3.45^***^			
		Problem-focused coping	−0.12	−0.11	−1.59			
		Emotion-focused coping	−0.09	−0.14	−1.96			
		Dysfunctional coping	0.00	0.00	0.03			
		Acceptance	−0.16	−0.49	−6.74^***^			
**Disability**	**1**	Educational level	−0.36	−0.16	−2.15^*^	4.64^*^	0.03	
	**2**	Educational level	−0.31	−0.14	−2.20^*^	39.73^***^	0.30	72.83^***^
		Pain intensity	0.74	0.55	8.53^***^			
	**3**	Educational level	−0.32	−0.15	−2.42^*^	25.17^***^	0.11	10.78^***^
		Pain intensity	0.70	0.52	8.65^***^			
		Problem-focused coping	−0.11	−0.10	−1.38			
		Emotion-focused coping	−0.04	−0.06	−0.91			
		Dysfunctional coping	0.24	0.30	5.05^***^			
	**4**	Educational level	−0.20	−0.09	−1.69	32.33^***^	0.11	38.89^***^
		Pain intensity	0.70	0.52	9.57^***^			
		Problem-focused coping	−0.10	−0.09	−1.43			
		Emotion-focused coping	0.01	0.02	0.32			
		Dysfunctional coping	0.07	0.09	1.35			
		Acceptance	−0.14	−0.41	−6.24^***^			
**PWB**	**1**	Employment^#^	−6.97	−0.20	−2.58^*^	6.65^*^	0.04	
	**2**	Employment^#^	−6.85	−0.19	−2.50^*^	3.34^*^	0.00	0.08
		Pain intensity	−0.12	−0.02	−0.28			
	**3**	Employment^#^	−4.69	−0.13	−1.98^*^	14.59^***^	0.27	21.27^***^
		Pain intensity	0.10	0.02	0.27			
		Problem-focused coping	1.00	0.23	2.95^**^			
		Emotion-focused coping	0.64	0.23	2.97^**^			
		Dysfunctional coping	−1.03	−0.33	−5.00^***^			
	**4**	Employment^#^	−5.07	−0.14	−2.51^*^	27.02^***^	0.19	61.93^***^
		Pain intensity	0.15	0.03	0.48			
		Problem-focused coping	0.90	0.20	3.11^**^			
		Emotion-focused coping	0.37	0.13	2.00^*^			
		Dysfunctional coping	−0.16	−0.05	−0.76			
		Acceptance	0.73	0.53	7.87^***^			

*PWB, Psychological Wellbeing*.

# *Dummy: 0 = employed; 1 = unemployed*.

* *p ≤ 0.05*.

** *p ≤ 0.01*.

*** *p ≤ 0.001*.

Coping strategies accounted for significant additional percentage of variance, which ranged from 11% (for disability) to 27% (for PWB) across the outcomes. The increments were moderate (0.46 for anxiety; 0.42 for depression; 0.33 for disability) to large (0.52 for PWB) (57). The contribution of psychological acceptance, entered in the last step, was statistically significant for all criteria, with increments in additional variance ranging from 11% (for disability) to 20% (for anxiety) over and beyond coping. These increments were moderate (0.45 for anxiety, 0.40 for depression, 0.33 for disability, and 0.44 for PWB).

In the last step of each equation, psychological acceptance proved to be a significant predictor for all the criteria: greater acceptance predicted lower anxiety, depression and disability, and higher PWB. However, the contribution of coping strategies to predict three of the four outcomes (i.e., anxiety, depression, and disability) was not statistically significant. The only exception was the regression model to predict PWB: both problem- and emotion-focused coping strategies positively predicted PWB in the last step of the equation.

### HMR models: Contribution of coping to explain adjustment (controlling for acceptance)

Table 3 shows the results of HRM models in which acceptance was entered before coping strategies. Psychological acceptance significantly increased the explained variance in all the outcomes, regardless of the contribution of background variables, with increments ranging from 21% (for disability) to 39% (for anxiety). The increment was large for anxiety (0.62), depression (0.54), and PWB (0.62), and moderate for disability (0.46).

**Table 3:** HMR models to predict adjustment (when acceptance is entered before coping).

**Criteria**	**Step**		***B***	**β**	***t***	***F***	**Δ*R*^2^**	***F*** **Change**
**Anxiety**	**1**	Pain intensity	0.47	0.30	3.99^***^	15.90^***^	0.09	
	**2**	Pain intensity	0.40	0.25	4.43^***^	74.30^***^	0.39	121.26^***^
		Acceptance	−0.25	−0.62	−11.01^***^			
	**3**	Pain intensity	0.38	0.23	4.16^***^	32.70^***^	0.03	3.09^*^
		Acceptance	−0.22	−0.55	−8.12^***^			
		Problem-focused coping	−0.08	−0.06	−0.90			
		Emotion-focused coping	−0.11	−0.13	−1.94			
		Dysfunctional coping	0.07	0.08	1.16			
**Depression**	**1**	Educational level	−0.37	−0.18	−2.35^*^	5.51^*^	0.03	
	**2**	Educational level	−0.35	−0.17	−2.27^*^	9.12^***^	0.07	12.36^***^
		Pain intensity	0.33	0.26	3.52^***^			
	**3**	Educational level	−0.24	−0.11	−1.87	34.71^***^	0.29	77.48^***^
		Pain intensity	0.28	0.22	3.65^***^			
		Acceptance	−0.17	−0.54	−8.80^***^			
	**4**	Educational level	−0.26	−0.13	−2.07^*^	20.67^***^	0.05	4.45^**^
		Pain intensity	0.27	0.21	3.45^***^			
		Acceptance	−0.16	−0.49	−6.74^***^			
		Problem-focused coping	−0.12	−0.11	−1.59			
		Emotion-focused coping	−0.09	−0.14	−1.96			
		Dysfunctional coping	0.00	0.00	0.03			
**Disability**	**1**	Educational level	−0.36	−0.16	−2.15^*^	4.64^*^	0.03	
	**2**	Educational level	−0.31	−0.14	−2.20^*^	39.73^***^	0.30	72.83^***^
		Pain intensity	0.74	0.55	8.53^***^			
	**3**	Educational level	−0.21	−0.10	−1.78	62.87^***^	0.21	74.13^***^
		Pain intensity	0.70	0.51	9.64^***^			
		Acceptance	−0.16	−0.46	−8.61^***^			
	**4**	Educational level	−0.20	−0.09	−1.69	32.33^***^	0.01	1.37
		Pain intensity	0.70	0.52	9.57^***^			
		Acceptance	−0.14	−0.41	−6.24^***^			
		Problem-focused coping	−0.10	−0.09	−1.43			
		Emotion-focused coping	0.01	0.02	0.32			
		Dysfunctional coping	0.07	0.09	1.35			
**PWB**	**1**	Employment^#^	−6.97	−0.20	−2.58^*^	6.65^*^	0.04	
	**2**	Employment^#^	−6.85	−0.19	−2.50^*^	3.34^*^	0.00	0.08
		Pain intensity	−0.12	−0.02	−0.28			
	**3**	Employment^#^	−6.13	−0.17	−2.86^**^	39.48^***^	0.38	107.45^***^
		Pain intensity	0.11	0.02	0.33			
		Acceptance	0.85	0.62	10.376^***^			
	**4**	Employment^#^	−5.07	−0.14	−2.51^*^	27.02^***^	0.08	8.90^***^
		Pain intensity	0.15	0.03	0.48			
		Acceptance	0.73	0.53	7.87^***^			
		Problem-focused coping	0.90	0.20	3.11^**^			
		Emotion-focused coping	0.37	0.13	2.00^*^			
		Dysfunctional coping	−0.16	−0.05	−0.76			

*PWB, Psychological Wellbeing*.

# *Dummy: 0 = employed; 1 = unemployed*.

* *p ≤ 0.05*.

** *p ≤ 0.01*.

*** *p ≤ 0.001*.

The contribution of coping strategies, when entered in the last step, was statistically significant for three out of four outcomes (i.e., anxiety, depression, and PWB), with increases in explained variance ranging from 3% (for anxiety) to 8% (for PWB). These increments were small (0.17 for anxiety, 0.22 for depression, and 0.28 for PWB). Moreover, coping explained an additional 1% of the variance in disability, but this increase was small (0.10) and did not reach the statistical significance.

## Discussion

Even though coping strategies have been extensively studied as predictors of adjustment to pain-related conditions (21, 22), including endometriosis (9, 39, 40), other psychological constructs have recently emerged to explain adaptation to pain. In particular, considerable research supports psychological acceptance as a relevant predictor of human functioning and behavioral effectiveness (34, 36, 37), but the evidence in the context of chronic pain is scarce and limited to samples of patients with mixed chronic pain conditions (35, 38). This study extended previous work to a sample of females with endometriosis recruited in a non-clinical setting.

The results showed that higher psychological acceptance was a significant predictor of lower anxiety, depression and disability, and higher PWB in this sample of females with endometriosis. Moreover, the inclusion of psychological acceptance in the regression models significantly improved the prediction of all outcomes over and beyond the effects of background variables, with moderate to large increments of variance. Overall, these results are consistent with previous related work supporting the criterion validity of psychological acceptance in chronic pain patients (35, 38). They also indicate that findings on the protective role of psychological acceptance are generalizable to nonclinical samples of females with endometriosis. Moreover, these findings further support the conceptualization of psychological acceptance as a general transdiagnostic process that is relevant to account for a number of mental health indicators across different contexts and populations (58, 59).

This study also expanded older related work (35, 38) by examining whether psychological acceptance explains unique variance in adjustment to endometriosis beyond the effects of coping. It is worth noting that most correlation coefficients for the relationship between coping and acceptance were weak, further corroborating acceptance as a construct that differs from the way people cope with stressful events as measured by the Brief COPE [e.g., (60–62)]. The results from the regression analyses evidenced incremental contributions of psychological acceptance to the variance of all four criteria (i.e., depression, anxiety, disability, and PWB) above and beyond coping strategies. In the more conservative regression models, in which coping was entered first, psychological acceptance demonstrated significant increments of variance averaging 17% (compared to 19% for coping). When the model was reversed, psychological acceptance predicted all the adjustment measures, with increments of variance averaging 32%, while the average increments of coping diminished to 4%.

Moreover, psychological acceptance did significantly predict all the criteria in the last step of each equation, whereas coping strategies failed to significantly predict three of the four outcomes in the last step. The only exception was the equation to predict PWB. Both greater problem- and emotion-focused coping were significantly associated with higher PWB. These findings are in line with previous studies reporting that acceptance of pain had greater utility than coping strategies in predicting functioning and distress in patients with chronic pain (23–25). Our results extend these earlier findings to general psychological acceptance, which accounted for more variance than coping strategies in four important measures of functioning.

Hence, taken all together, our findings show that the specific ways that females with endometriosis use to deal with pain and other stressors are less important than their willingness to experience pain and other unpleasant inner events (e.g., difficult feelings, memories, thoughts) without attempts to control them. Indeed, Vowles and McCracken (63) found that coping strategies did not correlate with improvements in functioning over the course of an interdisciplinary treatment for patients with chronic pain, while changes in acceptance processes did. As McCracken & Eccleston (23) have thoroughly argued, coping often refers to people's attempts to control stressors or unpleasant reactions to stressful situations. However, when stressors are uncontrollable (e.g., pain in chronic pain conditions) or unavoidable (as it often happens over the course of a lifetime), efforts aimed at controlling pain or other unwanted inner experiences (such as negative thoughts or feelings) are not useful. On the contrary, they may amplify psychological distress both because these internal events become more salient when people try to control them, and because efforts to avoid unpleasant inner experiences tend to narrow the range of behavior that are possible, since many behavior might indeed evoke unpleasant feelings and/or thoughts (34, 64–66).

Findings from this study have also important implications on psychological interventions in the context of pain. There is increasing empirical support for acceptance-based interventions for chronic pain such as ACT (26, 27). As mentioned above, ACT proved to be efficacious for enhancing functioning and decreasing psychological distress in patients with chronic pain [e.g., (28–30)]. While one of the main aims of ACT in chronic pain settings is enhancing acceptance of pain, as McCracken and Zhao O'Brien (35) claimed: “*there is more to accept than the pain itself* ” (p. 170). Psychological interventions may improve their effectiveness by fostering broader acceptance abilities that allow patients to accept not only pain, but also other difficult internal experiences. Indeed, females with endometriosis, in particular, and chronic pain sufferers, in general, often experience stressful events related not only to pain itself but also to other important life circumstances related to illness (e.g., illness-related infertility, couple difficulties or sexual dysfunctions in females with endometriosis). Willingness to experience the range of unpleasant thoughts and feelings that may arise from such stressful situations, beyond pain and without attempting to avoid them, can protect females for increased suffering over time (34, 35, 62).

Some limitations of this study should be highlighted. First, participants in this study completed a web-based assessment protocol. Several concerns have been raised about the quality of data collected through web-based surveys (67). Hence, future research could address whether the same results can be obtained by using other data collection methods. Our study also suffers from selection bias, since participation was voluntary and only AEP members were approached, and our sample size was small. Future studies could overcome these shortcomings to evaluate whether our findings can generalize. It should also be noted that coping strategies were measured by means of the Brief COPE in this study, but other operationalizations of coping exist (68). Further research is needed to examine whether psychological acceptance proves incremental validity over and beyond other conceptualizations of coping. The cross-sectional design of this study constitutes an additional limitation, since it impedes to infer cause-effect relationships between psychological acceptance and the outcomes measured in this study. Moreover, all the variables of this study were assessed by self-report methods, and this may raise concerns about the common method bias. Further studies are needed to explore the incremental validity of psychological acceptance when longitudinal design and other assessment tools are used.

Without neglecting the above limitations, the identification of psychological acceptance as a protective factor for quality of life, mental health and psychological wellbeing may be a useful finding for improving preventive and therapeutical approaches in the context of endometriosis. Overall, fostering psychological acceptance may contribute to reduce the personal, social and economic burden of pain and other illness-related stressors in females with endometriosis (32, 35, 38).

## Data availability statement

The raw data supporting the conclusions of this article will be made available by the authors, without undue reservation.

## Ethics statement

The studies involving human participants were reviewed and approved by Associazione Progetto Endometriosi. The patients/participants provided their written informed consent to participate in this study.

## Author contributions

OB and CBer contributed to conception and design of the study. OB and CBel collected data. OB, CBel, VC, LC, and GT organized the database. CBer performed the statistical analysis and wrote the first draft of the manuscript. CBer, OB, VC, LC, and GT wrote sections of the manuscript. All authors contributed to manuscript revision, read, and approved the submitted revision.

## Funding

Funder for open access publication fee: Department of Surgical, Medical and Molecular Pathology and Critical Care, University of Pisa, Italy.

## Conflict of interest

The authors declare that the research was conducted in the absence of any commercial or financial relationships that could be construed as a potential conflict of interest. The handling Editor MP declared a shared parent affiliation with the authors OB, GT, LC, VC, and CBer at the time of the review.

## Publisher's note

All claims expressed in this article are solely those of the authors and do not necessarily represent those of their affiliated organizations, or those of the publisher, the editors and the reviewers. Any product that may be evaluated in this article, or claim that may be made by its manufacturer, is not guaranteed or endorsed by the publisher.
