# Supplementary material for: Incremental validity of acceptance over coping in predicting adjustment to endometriosis
Source: Front Pain Res (Lausanne). 2022 Jul 15;3:928985. doi: 10.3389/fpain.2022.928985 (PMC9335002; doi:10.3389/fpain.2022.928985)
Supplement: Supplementary file 1 [file Data_Sheet_1.PDF]

Supplementary Material

1 Supplementary Figures

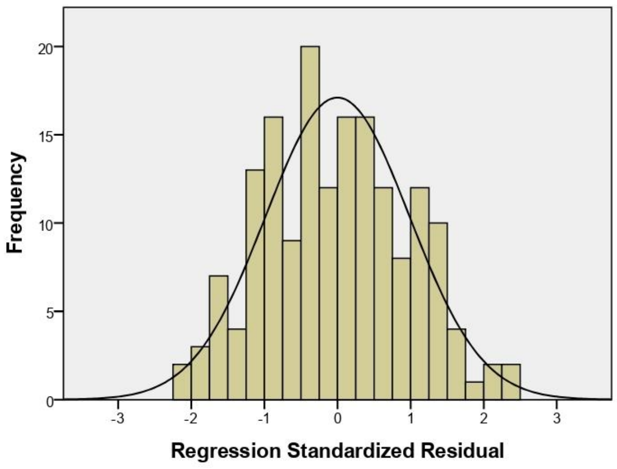

Supplementary Figure 1. Histogram of regression standardized residual for anxiety.

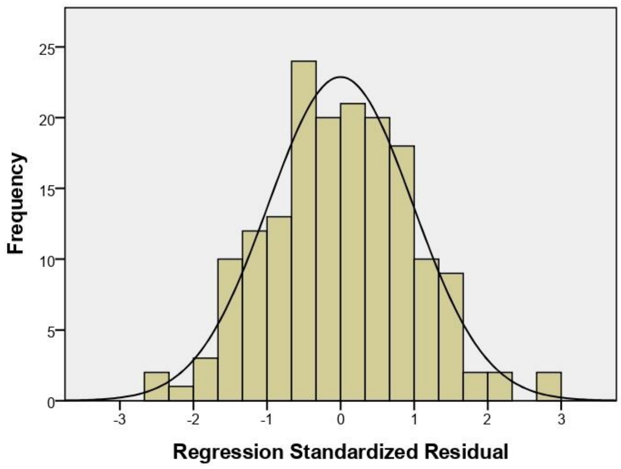

Supplementary Figure 2. Histogram of regression standardized residual for depression.

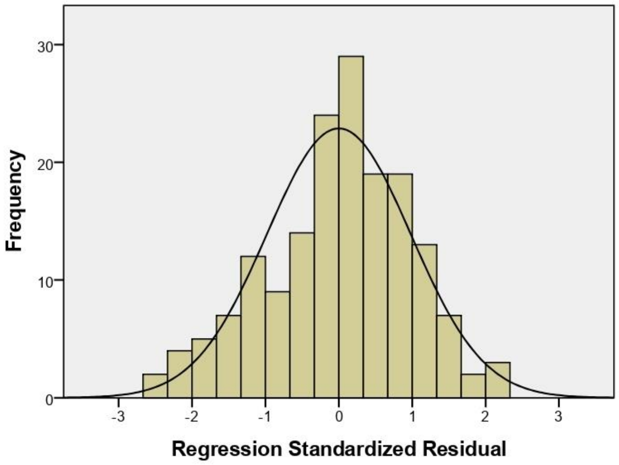

Supplementary Figure 3. Histogram of regression standardized residual for disability.

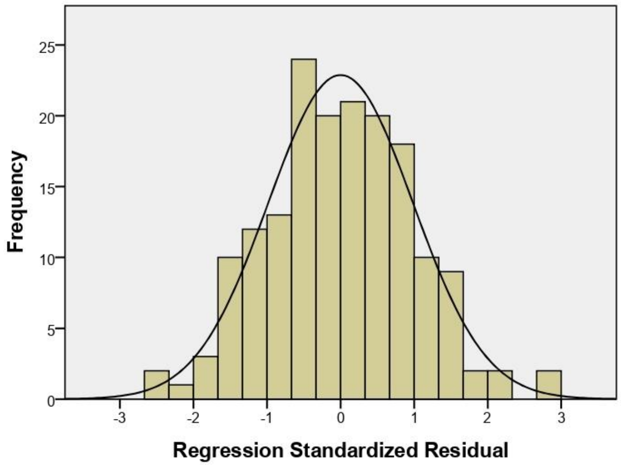

Supplementary Figure 4. Histogram of regression standardized residual for psychological well-being.

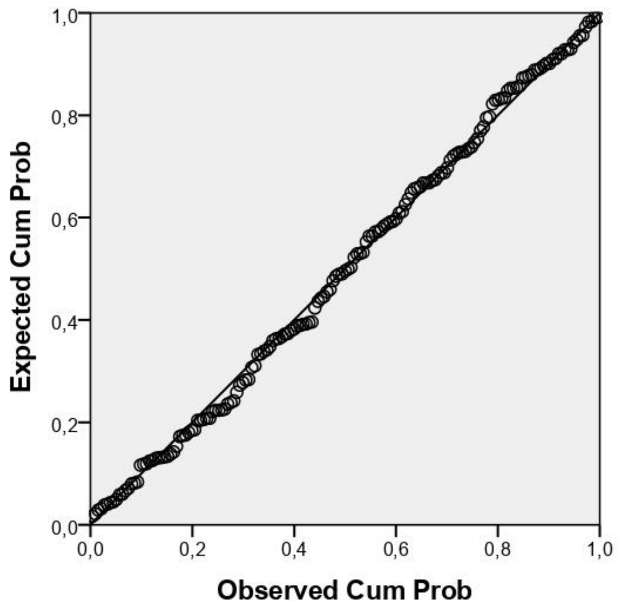

Supplementary Figure 5. Normal P-P plot of regression standardized residual for anxiety.

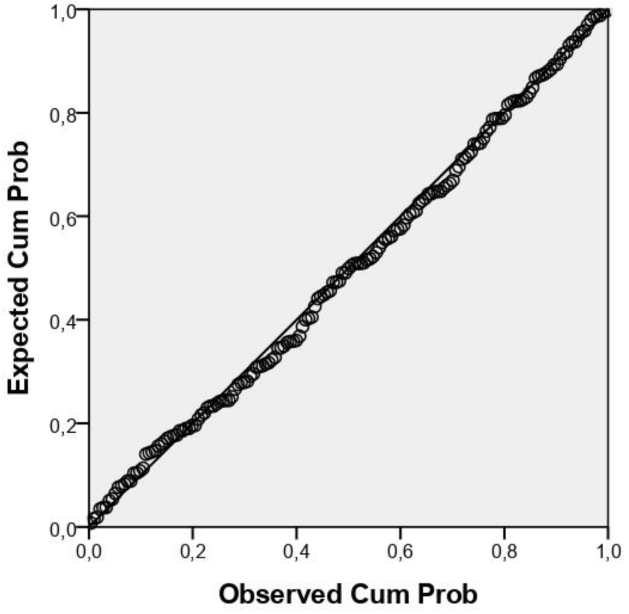

Supplementary Figure 6. Normal P-P plot of regression standardized residual for depression.

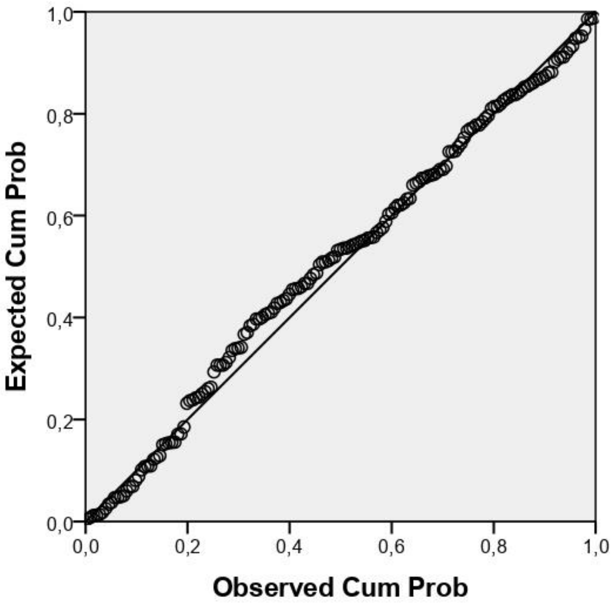

Supplementary Figure 7. Normal P-P plot of regression standardized residual for disability.

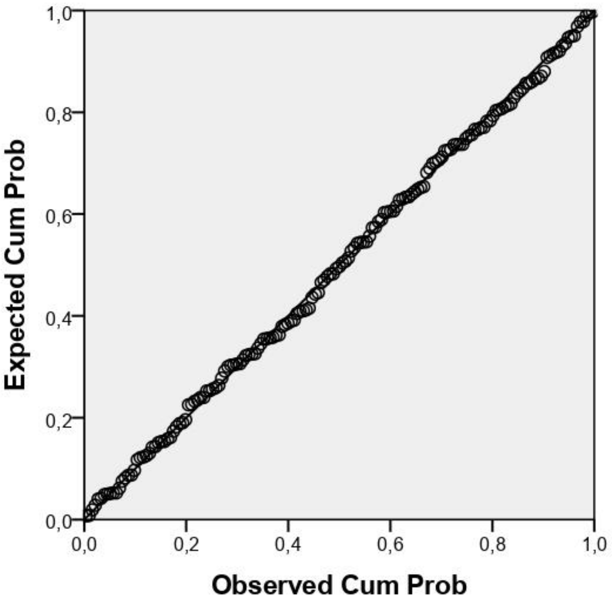

Supplementary Figure 8. Normal P-P plot of regression standardized residual for psychological well-being.

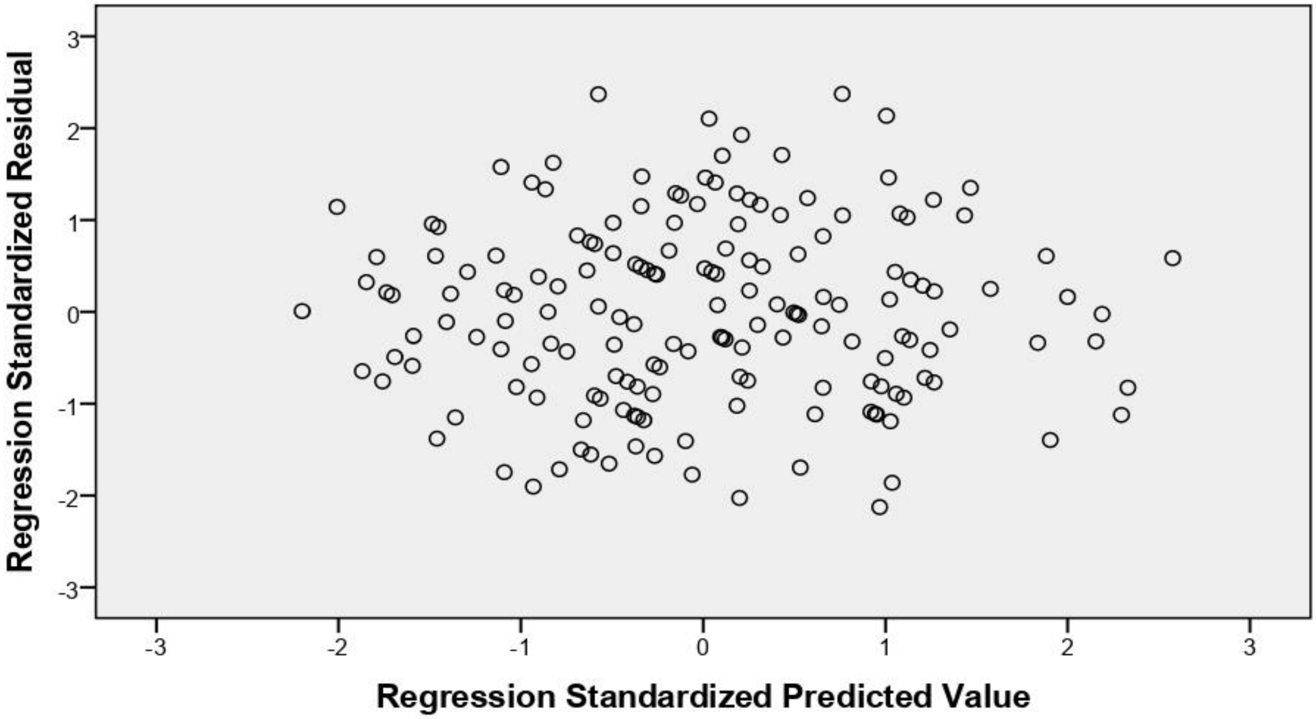

Supplementary Figure 9. Scatterplot of standardized residuals by standardized predicted values for anxiety.

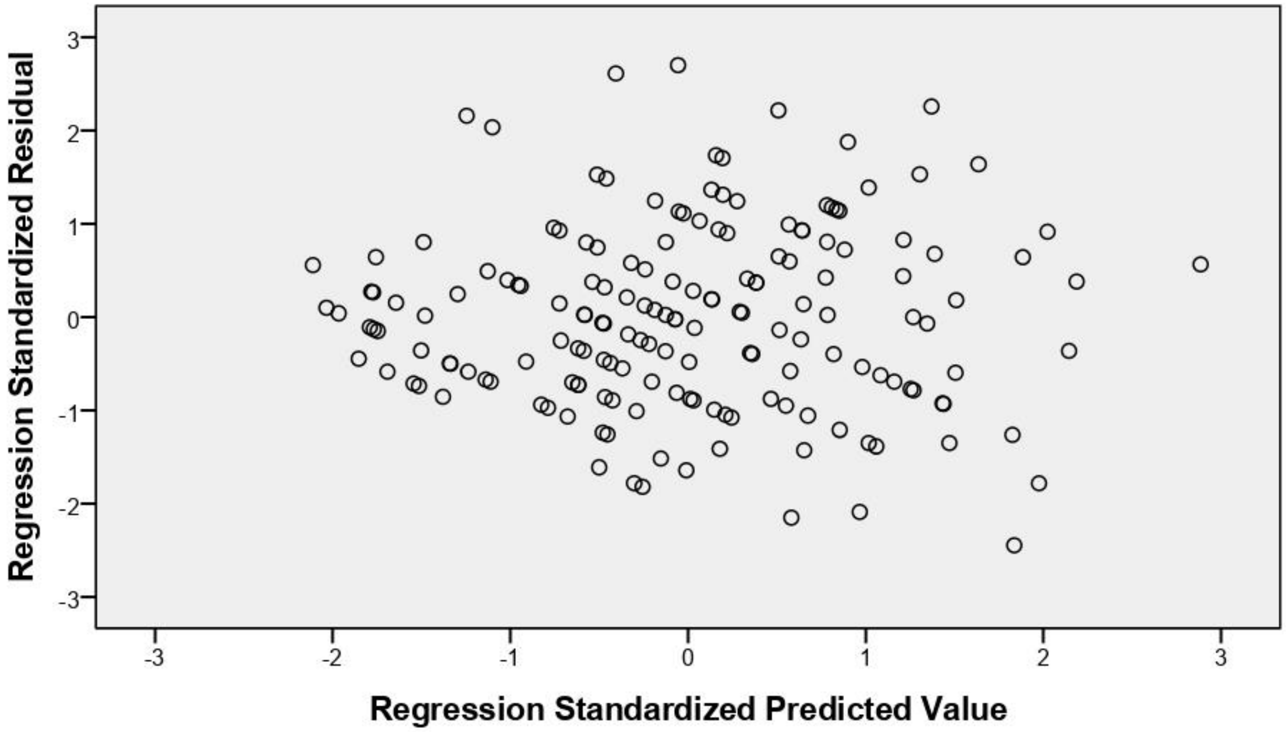

Supplementary Figure 10. Scatterplot of standardized residuals by standardized predicted values for depression.

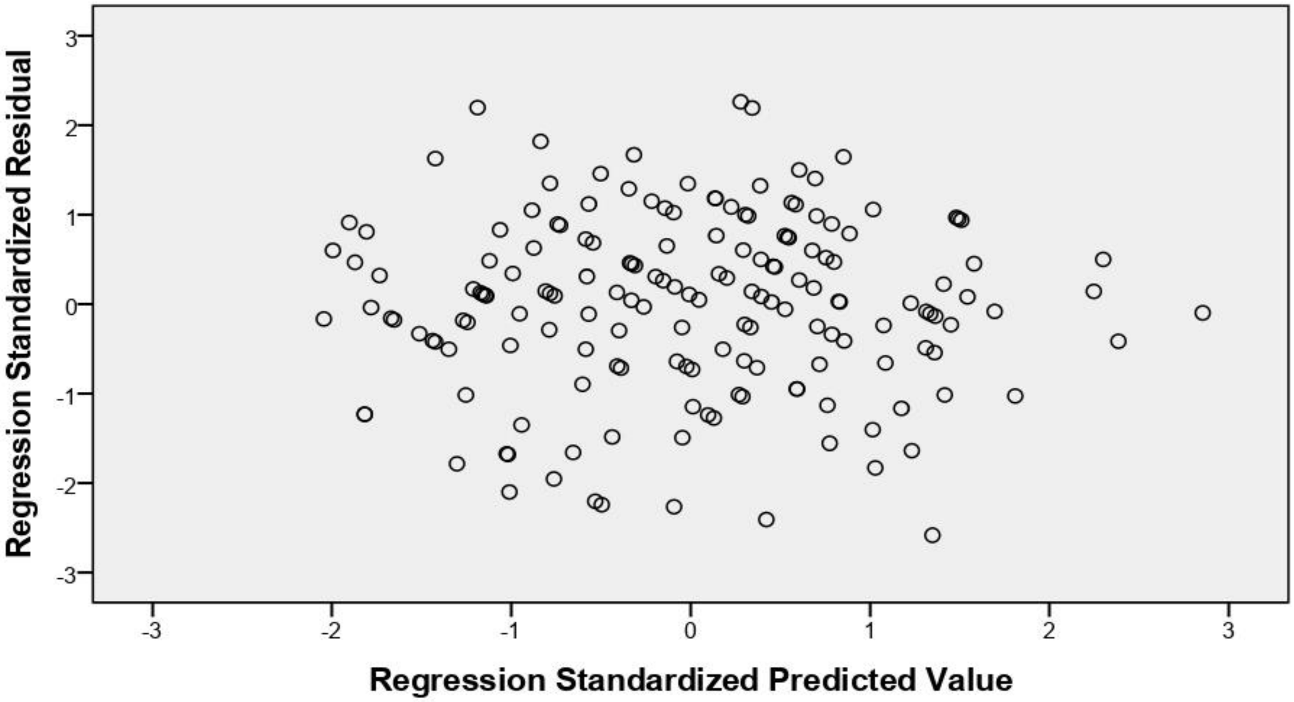

Supplementary Figure 11. Scatterplot of standardized residuals by standardized predicted values for disability.

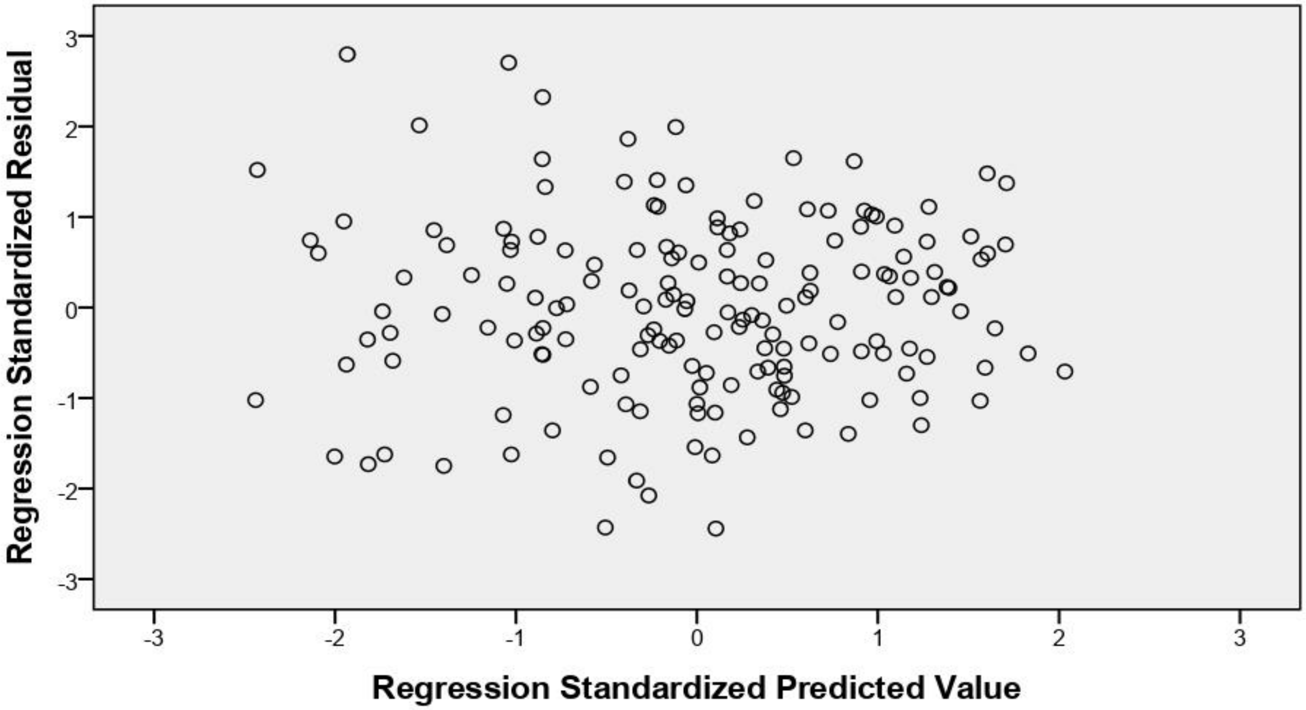

Supplementary Figure 12. Scatterplot of standardized residuals by standardized predicted values for psychological well-being.
